# Blood plasma metabolic profiling of pregnant women with antenatal depressive symptoms

**DOI:** 10.1038/s41398-019-0546-y

**Published:** 2019-08-23

**Authors:** Hanna E. Henriksson, Christina Malavaki, Emma Bränn, Vasilis Drainas, Susanne Lager, Stavros I. Iliadis, Fotios C. Papadopoulos, Inger Sundström Poromaa, George P. Chrousos, Maria I. Klapa, Alkistis Skalkidou

**Affiliations:** 1 Department of Women’s and Children’s Health, Uppsala University, Uppsala, Sweden; 2 Metabolic Engineering & Systems Biology Laboratory, Institute of Chemical Engineering Sciences, Foundation for Research and Technology-Hellas (FORTH/ICE-HT), Patras, Greece; 3 Department of Chemical Engineering, University of Patras, Patras, Greece; 4 Department of Neuroscience, Psychiatry, Uppsala University, Uppsala, Sweden; 5 First Department of Pediatrics, Athens University Medical School, Athens, Greece

**Keywords:** Diagnostic markers, Physiology

## Abstract

Antenatal depression affects ~9–19% of pregnant women and can exert persistent adverse effects on both mother and child. There is a need for a deeper understanding of antenatal depression mechanisms and the development of tools for reliable diagnosis and early identification of women at high risk. As the use of untargeted blood metabolomics in the investigation of psychiatric and neurological diseases has increased substantially, the main objective of this study was to investigate whether untargeted gas chromatography–mass spectrometry (GC–MS) plasma metabolomics in 45 women in late pregnancy, residing in Uppsala, Sweden, could indicate metabolic differences between women with and without depressive symptoms. Furthermore, seasonal differences in the metabolic profiles were explored. When comparing the profiles of cases with controls, independently of season, no differences were observed. However, seasonal differences were observed in the metabolic profiles of control samples, suggesting a favorable cardiometabolic profile in the summer vs. winter, as indicated by lower glucose and sugar acid concentrations and lactate to pyruvate ratio, and higher abundance of arginine and phosphate. Similar differences were identified between cases and controls among summer pregnancies, indicating an association between a stressed metabolism and depressive symptoms. No depression-specific differences were apparent among depressed and non-depressed women, in the winter pregnancies; this could be attributed to an already stressed metabolism due to the winter living conditions. Our results provide new insights into the pathophysiology of antenatal depression, and warrant further investigation of the use of metabolomics in antenatal depression in larger cohorts.

## Introduction

Antenatal depression, i.e., an episode of major depression during pregnancy^1^, affects ~9% of pregnant women in high income countries and more than 19% in low- and middle-income countries^2^. Apart from the suffering of the women, the condition can also have adverse consequences such as preterm birth, low birth weight, and altered behavioral development of the offspring^3,4^. Antenatal depression is considered a complex multifactorial disease, as a combination of biological and environmental factors seem to contribute to its onset^5,6^. Decreased levels of serum allopregnanolone^7^, morning cortisol^8^, and oxytocin^9^ have been reported among depressed pregnant women. Similarly, a disrupted immune response, with both decreased and increased levels of inflammatory markers has been reported among women with antenatal depression, when compared with controls^10–13^. However, the identified differences in the measured parameters have not led to the development of reliable diagnostic tests. It is, therefore, critical to further investigate the pathophysiology of antenatal depression and its underlying biological mechanisms, in a holistic, discovery-driven way. Using the omic analyses of the systems biology era to identify biomarkers with sufficient accuracy and sensitivity for early disease diagnosis and/or the identification of women with higher disease susceptibility, may facilitate the development of preventive measures and more targeted effective treatment.

Untargeted blood plasma metabolomics, i.e., the analysis of the concentration profile of the free small molecular-weight metabolites in the blood plasma, also known as the blood plasma metabolic profile, has been deployed in biomedical applications in the context of disease diagnosis^14^. Investigating the metabolic physiology may provide insights to the genetic and epigenetic fingerprints of an individual. Further, untargeted metabolomics may aid in the investigation of molecular disease mechanisms, as well as the design of novel drugs and appropriate treatments^15^. Metabolomics has previously been used in psychiatry research to study, e.g., metabolic profile in relation to major depressive disorder and posttraumatic stress disorder^16–19^, however, among women experiencing depression or depressive symptoms during the peripartum period the literature is scarcer. A study of urine metabolomics in postpartum depression identified a panel of five biomarkers (formate, succinate, 1-methylhistidin, α-glucose, and dimethylamine), which could distinguish postpartum depressed from postpartum non-depressed women, as well as from healthy controls (area under curve (AUC) in training set = 0.948 and in testing set = 0.944)^20^. In addition, a recent metabolomics study identified ten metabolites that had an altered abundance among postpartum depressed women compared with healthy controls^21^. However, to date, there are no published studies on untargeted blood metabolomics among pregnant women with depressive symptoms.

Seasonal variations in the metabolism have been suggested^22–24^. In addition, the metabolism has been linked to the immune function^25,26^, and previous studies in the general population have reported a seasonal pattern in gene expression and components of the immune system^27–31^. Dopico et al.^27^ reported an increased pro-inflammatory transcriptomic profile when analyzing samples from European populations in the winter vs. the summer. This rise was coupled with an increase in circulating C-reactive protein (CRP) and the soluble interleukin (IL)-6 receptor^27^, markers that have been linked to depression^32,33^. Although results were inconclusive regarding whether depressive symptoms vary with season in peripartum populations^34–37^, studies have indicated winter as the risk season. Furthermore, studies have reported on the association between the immune system and metabolism^25,26,38,39^. Thus, seasonal variation should be investigated as a potentially differential parameter, when analyzing the blood metabolic profile of the pregnant women with and without depressive symptoms.

In this context, the aims of this study were to investigate: (i) whether the plasma metabolic profile is discriminatory between pregnant women with and without depressive symptoms, and (ii) whether there are seasonal variations in the metabolic profiles of these groups when categorized into summer and winter childbirths.

## Materials and methods

### Selection of the cohort

This cross-sectional study was undertaken as part of the BASIC (Biology, Affect, Stress, Imaging, and Cognition) project, an on-going population-based panel study investigating correlates of peripartum affective symptoms among women giving birth at Uppsala University Hospital, Uppsala, Sweden^40,41^. Women residing in Uppsala County, who register for the routine ultrasound examination around gestational week 17, are asked about participation via postal mail. Exclusion criteria are age younger than 18 years, protected identity, inability to communicate adequately in Swedish, blood-borne infectious diseases, and non-viable pregnancies. The data collected were mainly derived from web surveys, which include the Edinburgh Postnatal Depression Scale (EPDS). The EPDS is a self-administered psychometric questionnaire used to identify depressive symptoms during pregnancy and after childbirth^42^. It consists of ten statements concerning events that have occurred during the last seven days, and each statement has four alternative answers with a score from 0 to 3. In 2012, the Swedish Council on Health and Technology Assessment^43^ published a systematic review of diagnostics and follow-up of affective disorders. In their report, they concluded that there were too few studies on antenatal depression to recommend any cut-off for the usage of the EPDS during pregnancy. The Swedish validation recommends an EPDS score cut-off of ≥13 points, with a sensitivity of 77% and a specificity of 94%^44^. Nevertheless, to avoid compromising the sample size of the current study, an EPDS score cut-off of ≥12 points was used to distinguish women with depressive symptoms from those without. This cut-off is supported by a study using the non-patient version of the Structural Clinical Interview for DSM-III-R (SCID-NP) as a reference among pregnant women^45^.

### Sample-size estimation

For this study, 50 samples were selected from a subgroup of women participating in the BASIC project, described above. The total sample size of 50 was selected based on a number of initial investigatory metabolomics studies, which could indicate whether there is a significant trend in the data, in order to be then able to design larger epidemiological studies. It needs to be noted that in the case of metabolomics, large epidemiological studies are currently limited and there has been a large discussion about all the pre-analytical steps (e.g., sample collection, handling, aliquoting etc.), which we have investigated in the present study and have taken into consideration. Owing to the relatively small total number of samples, we made efforts to have these as homogeneous as possible with respect to parameters that may affect the metabolic profile apart from the antenatal depressive symptoms, such as fasting status and season. For this reason, women that underwent cesarean section and were all after overnight fasting were included, and grouped by season of childbirth. Furthermore, range criteria on, e.g., age and Body Mass Index (BMI) were applied, as described below.

### Sample description

The participants included in this sub-study were women who would undergo an elective cesarean section, as they were fasting for at least 12 h before blood sampling, which is very important for the planned analyses (Supplementary Table 1). The selected samples had been collected from 26 to 39 years old participants who provided a blood sample prior to an elective cesarean section at around gestational week 38. For the current study, exclusion criteria were age beyond the selected range, BMI < 21 or >39 kg/m^2^, parity ≥ 4, twin pregnancy, smoking, and a hypertension or diabetes diagnosis. These criteria were selected as they might affect the quality of the MS profiles and the biological diversity that can be observed by metabolic profiling. They are strict in order to ensure high quality of the data finally included in the analysis. The EPDS was answered few days before childbirth; when that data was not available, EPDS data from gestational week 32 was used instead. Thirty-two participants with an EPDS score between 1 and 8 were considered controls and 18 participants who scored between 12 and 30 were considered cases. Participants with an EPDS score between 9 and 11 were excluded, in order to generate two clearly separate groups, aiming at decreasing misclassification in the outcome. Furthermore, to allow for investigation of any seasonal variation, women were divided in two seasonal groups according to the date of childbirth. Births between March 21 and September 20 were considered as occurring in “summer (S)” (*n* = 17), and those between September 21 and March 20 as “winter (W)” births (*n* = 28). The dates were chosen based on the spring and fall equinoxes.

At gestational week 17, information on educational attainment, previous depression, smoking, weight, and height was collected. The final variable on previous depression included information on self-reported previous depression and/or a visit to a psychiatrist or psychologist. At gestational week 32, the participants stated current employment status and whether they were suffering from any of the following pregnancy complications: gestational hypertension, gestational diabetes, and/or preeclampsia. From the medical journals, data on any pregnancy complication, the date of childbirth and any medication used, were gathered.

### Ethical considerations

The study protocol has been approved by the Regional Ethical Review Board of Uppsala, Sweden (Dnr 2009/171). Written informed consent was obtained from all participants when entering the BASIC study, as well as before undergoing elective cesarean section, prior to any sampling or testing.

### Sample collection

Collection of fasting blood samples in EDTA-smeared plasma collection vacutainer (Vacuette®, Hettich Labinstrument) was carried out just before the cesarean section. Samples were centrifuged at 1500 RCF (relative centrifugal force) at room temperature for 10 min, then the plasma was transferred to a new tube and stored at −70 °C until analysis. Aliquots of 100 μL were collected for each sample and two aliquots per sample were sent to the Institute of Chemical Engineering Sciences, Foundation for Research and Technology-Hellas (FORTH/ICE-HT), Patras, Greece, in dry ice for the metabolomic analysis.

### Metabolomic data acquisition and normalization

The analysis was carried out at the Metabolic Engineering & Systems Biology Laboratory of FORTH/ICE-HT, using gas chromatography–mass spectrometry (GC–MS) for the acquisition of the polar metabolite profiles. The laboratory personnel performing the MS profiling was unaware of the case-control status and other characteristics of the samples. Thus, during the analyses, there was completely blind selection of the samples to be quantified for their metabolic profile with respect to the various groups. Extraction and GC–MS metabolic profile acquisition protocols have been previously described^46,47^. To each 100 µL sample aliquot, 0.05 μg ribitol (Alfa Aesar, Germany) and 1 μg [U-^13^C]-glucose (Cambridge Isotope Laboratories, USA) were added as internal standards. The dried extract of each aliquot was derivatized to its (MeOx)TMS-derivatives through reaction with 50 μL of 20 mg/mL methoxyamine hydrochloride (Alfa Aesar, Germany), in pyridine (Carlo Erba Reagents, Italy) for 90 min, followed by reaction with 100 μL *N*-methyl-trimethylsilyl-trifluoroacetamide (MSTFA) (Alfa Aesar, Germany) at 40 °C for at least 6 h. The metabolic profile of each aliquot was measured at least thrice at different derivatization times using a Saturn 2200 ion-trap GC–MS (formerly Varian Inc., now Bruker (GC)/Agilent (MS)). The peak identification and quantification was based on the commercial NIST and our in-house MESBL peak library. A total peak area for glucose was estimated as the sum of glucose-MeOx1, glucopyranose 1, and glucopyranose 2 derivative marker ion peak areas and this was used in further analysis. The metabolic profile data validation, normalization, and filtering were carried out using the M-IOLITE software suite (http://miolite2.iceht.forth.gr)^48–50^, estimating the relative peak areas (RPAs) of the marker ions of each metabolite derivative with respect to the peak area of the ribitol ion 217. Metabolites that had a mean coefficient of variation (CoV) between injections/profiles of the same sample larger than 25% were filtered out; the same applies for the biological replicates. After filtering, the normalized profiles comprised 38 metabolites. Paracetamol was detected in few samples with two samples containing considerable concentrations (relative to the median abundance of the other molecules); the presence of paracetamol was considered as complementary information and was not included in the subsequent multivariate analysis of the profiles. The metabolic profile of each aliquot was estimated as the mean of the normalized profiles of all its technical replicates. The metabolic profile of each sample was estimated as the mean metabolic profile of its aliquots. Three samples, one case and two controls, did not yield sound metabolic profiles and were excluded from further analysis (Supplementary Table 1). The final normalized metabolic profile dataset (including the paracetamol measurements) of the 47 samples is shown in Supplementary Table 2.

A principal component analysis (PCA) of the metabolic profiles indicated two women, both cases but from different seasonal groups, as having a substantially different profile than all others (Supplementary Fig. 1). Owing to the observed large differences with respect to the other samples, which pointed to additional medical issues (e.g., chorioangioma), these two samples were excluded from further analyses. This resulted in a final dataset of 37 metabolite profiles from 45 plasma samples, 15 from cases and 30 from controls, considered in this study for the extraction of biologically relevant conclusions.

### Statistical analyses

#### Demographic, medical, and questionnaire data

Any differences in the demographic, medical, and questionnaire data between the four groups were evaluated by non-parametric tests, as the data were not normally distributed. The Kruskal–Wallis test was applied to examine associations between continuous variables. Likewise, the Fisher’s exact test (two-sided) was applied to examine associations between categorical variables. A *p*-value of <0.05 was considered as statistically significant. Analysis was conducted using SPSS version 24 (IBM Corp, Armonk, NY) and the STATA-9 statistical software.

#### Metabolomic dataset multivariate statistical analysis

Hierarchical clustering (HCL), PCA, and significance analysis of microarrays (SAM) algorithms were applied as implemented in version 4.8.1 of the omic data analysis TM4 MeV software^51^. The analysis was carried out with missing values not imputed. The metabolites with concentration significantly higher or lower in a set of metabolic profiles compared to another, were, respectively, referred to as positively or negatively significant metabolites of the particular comparison. Where standardized RPA values are mentioned, the standardized RPA of metabolite M in profile j,$\,st{\text{RPA}}_{_{\text{M}}}^{\text{j}}$, is equal to:

$$\,st{\text{RPA}}_{_{\text{M}}}^{\text{j}}={\frac{{\text{RPA}}_{_{\text{M}}}^{\text{j}}-{\overline {{\text{RPA}}_{^{\text{M}}}}}}{{\text{SD}}_{{\text{RPA}}_{\text{M}}}}}$$

where ${\text{RPA}}_{_{\text{M}}}^{\text{j}}$, $\overline {{\text{RPA}}_{^{\text{M}}}}$, ${\text{SD}}_{{\text{RPA}}_{\text{M}}}$ depict, respectively, the RPA of metabolite M in profile j, the mean RPA of metabolite M in all profiles and its standard deviation. In SAM, the threshold of significance is selected as the largest for the false discovery rate (FDR)—median to be smaller than 10%. This computational analysis has previously been described for untargeted GC–MS metabolomics in Papadimitropoulos et al.^47^.

## Results

The clinical characteristics of the study sample are presented in Table 1. One summer case and one winter case were on treatment with selective serotonin reuptake inhibitors (SSRIs) in gestational week 32. According to their medical journals, one woman was on treatment prior to the current pregnancy, while the other one initiated treatment around gestational week 30, both because of depression. Around half of the winter controls reported previous depression, while the number was 67% among summer controls and 86% among both summer and winter cases. The median time between the gestational week 32 survey and date of childbirth was 6.57 weeks (Interquartile range, IQR = 6–7).

**Table 1:** Background characteristics of the participants (*n* = 45)

	Summer control	Summer case	Winter control	Winter case
Participants, *n*	10	7	20	8
Age in years, median (IQR)	34.0 (30.0–35.3)	34.0 (28.0–35.0)	34.5 (33.0–35.0)	30.5 (28.5–33.8)
Pre-pregnancy BMI, median (IQR)	23.8 (21.9–25.0)	24.3 (23.0–27.8)	24.5 (22.5–26.1)	23.7 (22.7–24.3)
EPDS score, median (IQR)	6.0 (4.8–8.0)	14.7 (13.0–16.0)	3 (2.0–5.6)	15.5 (14.0–16.8)
EPDS assessed prior to cesarean section, *n* (%)	2 (20.0)	4 (57.1)	6 (30.0)	3 (37.5)
EPDS assessed in gestational week 32, *n* (%)	8 (80)	3 (42.9)	14 (70.0)	5 (62.5)
Levothyroxine treatment, *n* (%)	1 (10.0)	1 (14.3)	0 (0.0)	2 (25.0)
SSRI treatment, *n* (%)	0 (0.0)	1 (14.3)	0 (0.0)	1 (12.5)
Previous depression^a^, *n* (%)	6 (66.7)	6 (85.7)	9 (47.4)	6 (85.7)
Nulliparous, *n* (%)	2 (20.0)	2 (28.6)	5 (25.0)	2 (25.0)
Gestational age in days, median (IQR)	273.5 (270.0–276.3)	273.0 (272.0–275.0)	272.5 (270.3–277.0)	272.0 (268.0–273.8)

Statistical differences measured by Kruskal–Wallis test and Fisher’s exact test between the four groups

*IQR* interquartile range, *BMI* body mass index, *EPDS* Edinburgh postnatal depression scale, *SSRI* selective serotonin reuptake inhibitors

^a^Missing values affect percentages

### Metabolomic data analysis

**Fig. 1:**
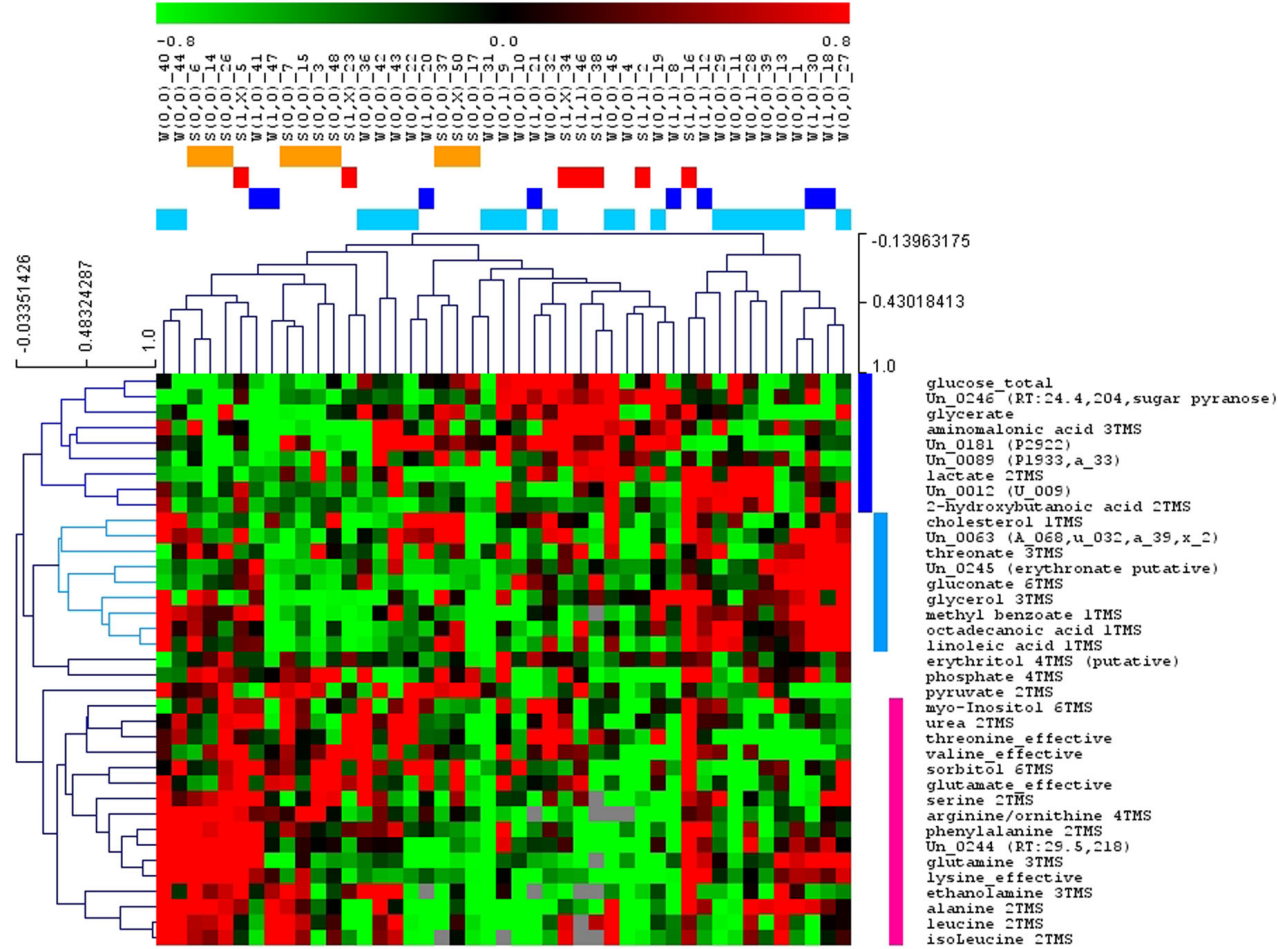
**The hierarchical tree resulting from the hierarchical clustering analysis of the standardized GC–MS metabolic profiles (Pearson correlation coefficient distance metric).** The samples of the summer controls S(0), summer cases S(1), winter controls W(0), and winter cases W(1), are colored orange, red, light and dark blue, respectively (see on top of the tree). S(*xx*,*yy*) and W(*xx*,*yy*) depict antenatal summer or winter cases, respectively, if *xx* = 1, and controls if *xx* = 0. If the postpartum condition of the women is known, then those with depressive symptoms are depicted with *yy* = 1 and 0 otherwise. *X* indicates an unknown postpartum condition for these women. The number at the end of the sample name corresponds to the sample no. in Supplementary Table 1. Three main metabolite clusters are indicated colored in dark blue, light blue, and pink (see right side of the heatmap)

Figure 1 and Supplementary Fig. 2 show, respectively, (a) the hierarchical trees of the plasma samples and the metabolites resulting from the HCL and (b) the graph of the metabolic profiles from the PCA of the metabolomic dataset (45 samples). The hierarchical tree of the metabolites revealed three main groups with respect to their concentration profiles (Fig. 1). The first group comprised samples with high levels of glucose, a sugar pyranose, glycerate, lactate, aminomalonic acid, and 2-hydroxybutanoic acid. The second group included cholesterol, the polyunsaturated fatty acids (PUFAs) octadecanoic and linoleic acid, methyl-benzoate, glycerol, threonate, gluconate, erythronate, phosphate, and erythritol. The third group included all detected amino acids, myo-inositol, urea, sorbitol, and pyruvate, with myo-inositol, urea, threonine, valine, glutamate, sorbitol, and pyruvate forming a separate subgroup from the rest of the molecules (all amino acids). A clustering of the metabolic profiles based mainly on these three groups is apparent from the respective hierarchical tree (top of the heatmap in Fig. 1). PCA supports the observation from HCL (Supplementary Table 3), indicating a separation of the profiles in the principal component 1 (PC1) axis based on the concentration of glucose and the unknown sugar pyranose (high on the positive PC1/low on the negative) and of lysine, arginine, phenylalanine, and serine (low on the positive PC1/high on the negative). Moreover, profiles with positive PC2 values are rich in metabolites associated with the lipid-enriched second group identified from HCL analysis, but with lower concentrations of valine and threonine. Finally, positive PC3 values indicate mainly profiles with high concentrations of leucine, isoleucine, alanine, and pyruvate, and lower abundance in myo-inositol, sorbitol, and gluconate.

Multivariate significance analysis, SAM, was applied to identify the metabolites that are actually of statistically differential abundance between the various groups of interest, complementing the findings from HCL and PCA. Comparing the cases to the controls independently of the season of birth, no significant differences in their metabolic profiles were observed. However, the summer cases and controls presented diverse metabolic profiles. The discriminatory metabolites, identified by SAM, are shown in Table 2 and are supported by HCL (Fig. 1) and PCA (Supplementary Fig. 2). The majority of the summer cases were characterized by higher abundance of the first (glucose-rich) and second (lipid-rich) metabolite clusters, and a lower abundance of the third (amino acid-rich) metabolite cluster. In the PCA graph, these profiles correspond mainly to positive PC1 and PC2, and negative PC3.

**Table 2:** Metabolites with differential abundance in the summer controls compared with the summer cases

Higher abundance in summer controls	Lower abundance in summer controls
	1. un_0246 (RT:24.4,204,sugar pyranose)
2. Phosphate	
3. Arginine	
FDR—median = 0%	
	4. un_0245 (erythronate putative)
	5. Urea
	6. un_0012 (U_009)
	7. Aminomalonic acid
	8. Glycerate
	9. Glucose
	10. Threonine
	11. Gluconate
FDR—median = 4.9%	
	12. Lactate
13. Pyruvate	
FDR—median = 8.32%	

The metabolites are shown in decreasing order of statistical significance in SAM for a false discovery rate (FDR)—median = 8.32% (i.e., 1 potentially false-positive metabolite out of the 13 identified); the results at stricter FDR thresholds are also indicated. The unknowns are shown with their number in the in-house metabolomic peak library (in parenthesis we include labels that have been used in previous publications for comparability purposes)

Moreover, significant differences were observed between summer and winter controls. The statistically differential metabolites identified by SAM are shown in Table 3. Interestingly, the summer case profile resembles largely that of the winter control, as it is also supported by HCL and PCA results. There was no clear discrimination between the metabolic profiles of the cases and the controls among the winter samples, nor between samples from summer and winter cases, as evident by SAM and also supported by the HCL and PCA analyses.

**Table 3:** Metabolites with differential abundance in the winter controls compared with the summer control samples

Higher abundance in winter controls	Lower abundance in winter controls
	1. Arginine
	2. Phosphate
	3. Pyruvate
FDR—median = 0%	
4. un_0246 (sugar pyranose)	
5. Aminomalonic acid	
6. un_0012 (U_009)	
7. un_0245 (erythronate putative)	
8. Gluconate	
FDR—median = 9.46%	

The metabolites are shown in decreasing order of statistical significance in SAM for a false discovery rate (FDR)—median = 9.46% (i.e., < 1 false positive metabolite out of the 8 identified); the results at the strictest FDR = 0% threshold are also indicated. The unknowns are shown with their number in the in-house metabolomic peak library (in parenthesis we include labels that have been used in previous publications for comparability purposes)

## Discussion

In this study, we demonstrate the use of untargeted metabolomics in discriminating plasma metabolite profiles between pregnant women with and without depressive symptoms. In the overall analysis, comprising samples of both summer and winter childbirths, no significant differences in the metabolic profiles between cases and controls were observed. Nevertheless, we demonstrate that there is a discriminatory metabolic profile between cases giving birth during the summer and their respective controls. Furthermore, we demonstrated that the metabolic profiles in late pregnancy differ between controls giving birth in the summer compared with those giving birth in the winter.

Among the summer samples, we identified a higher abundance of glucose and lactate and a lower abundance of pyruvate in cases than in controls. The higher abundance of glucose combined with the higher lactate to pyruvate ratio indicate alterations in both the tricarboxylic acid (TCA) and the Cori cycles which have been associated with metabolic stress conditions^46^. Lin et al.^20^, reported also significant lactate abundance differences in the urine metabolomic study among postpartum depressed women, compared with both postpartum controls and non-pregnant controls.

Plasma arginine, an amino acid, has previously been reported lower among women with antenatal depression in the first trimester, when compared with healthy controls^52^. In a recent untargeted metabolomics study, tyrosine was identified in lower abundance in urine among postpartum depressed women when compared with healthy controls^21^. On the contrary, the concentration of alanine and homocysteine were reported to be elevated among postpartum depressed^21^.

Aminomalonic acid was found in higher abundance in both summer cases and winter controls, when compared with summer controls. It is a metabolite possibly originating from errors in protein synthesis, and has been associated with oxidative stress conditions^53^. Summer cases, in comparison with summer controls, also had a statistically higher abundance of glycerate than the other two groups. Glycerate shares a pathway with oxalate, with increased concentration of the latter being associated with increased lipid β-oxidation^54^. Moreover, 2-hydroxybutanoic acid is an early indicator of insulin resistance in non-diabetic subjects, and has been suggested as a predictor of glucose intolerance progression^55^.

The higher abundance in stress-associated metabolites identified in most summer cases and winter controls in relation to the summer controls has to be considered in combination with the observed lower concentration in essential amino acids (e.g., threonine and the branched chain amino acids (BCAAs) leucine, and isoleucine) and myo-inositol. BCAAs were recently proposed as biomarkers of depression and their observed profile in our study is in agreement with these reports^56^.

With respect to seasonal variation, the profiles of the winter controls suggest winter as a season with a metabolic risk profile, in line with other studies^22,24^. On the contrary, the majority of women in the summer control group had amino acid-rich profiles with a lower abundance in sugars and/or PUFAs, suggesting a healthier cardiometabolic profile. In Sweden, the winter is characterized by prolonged darkness and cold temperatures, which could be regarded as an environmental stressor. These observations could partially be due also to the differences in diet between the seasons, e.g., an increased consumption of salads and fresh vegetables during summer compared to winter. Unfortunately, no information on the participants’ diet was collected to conclusively interpret our observations. However, this study forms the basis for the extension of the questionnaire to diet information, while supporting the seasonal variation which could be an additional risk factor for antenatal depression.

Strengths of this study include its novelty with respect to the application of untargeted blood plasma metabolomics in antenatal depression and the suggestion of biomarker profiles for summer cases and between seasons. Untargeted metabolomics is unbiased in the sense that the metabolites have not been subjectively chosen based on an original hypothesis, thus can provide new insights regarding the particular pathophysiology. A robust quality control process ensured appropriate profile normalization and correction from any experimental biases. The main limitation of the study was the sample size, which preferably should be larger than 12 participants per group, and was thus planned. The number of samples in some subgroups ended up being smaller. This was primarily because of last minute unavailability of subjects to be recruited in the sub-study during certain periods, and other administrative reasons; the exact number of subjects within each subgroup was made clear after the categorization and technical control of all samples. The inadequate sample size might have compromised a more thorough analysis of differences between winter cases and winter controls, if these indeed exist. The identification of participants based on a screening tool and not a psychiatric interview has also to be considered. The included cases would probably not all have fulfilled diagnostic criteria for depression, due to moderately elevated EPDS scores. The discrepancy between the time-point of EPDS administration and time of sampling is another limitation that has to be acknowledged. All women were administered the questionnaire prior to the cesarean section, but only a minority (15/45) completed it at that time-point, therefore their classification as case or control was based on their score in gestational week 32, taking place ~7 weeks earlier. This limitation may bias the results if the women would have switched depressive symptoms status during this time-period; nevertheless, the strong correlation between EPDS scores at weeks 32 and 38 observed in the whole of the BASIC cohort, as well as the exclusion of women with scores 9, 10, and 11 might indicate that the degree of misclassification is expected to be significant. Another limitation that has to be considered is that history of depression was higher among cases of antenatal depressive symptoms but quite prevalent even among controls. If depression has long-term effects on biological systems, this may limit the ability to find differences between the groups, or even that differences found among mothers with summer childbirths might be because of history of previous depression and not just current antenatal depressive symptoms. Moreover, one woman from summer and one among winter cases reported treatment with SSRIs at gestational week 32 due to depression, and both were on treatment at the time of blood sampling; how this treatment may affect the metabolic profile and thus our results in not known. With regard to the definition of season, due to the limited sample size, it was not possible to create more clear-cut seasons, such as only including births in, e.g., June–August and December–February. Finally, no information regarding the women’s diet in general was available, and, thus, it was not possible to verify major differences in diet between seasons. Acute dietary standardization was reported to decrease the variation of urinary metabolic profiles, but not in plasma or saliva^57^. The finding of paracetamol in many samples is not surprising as it is generally administered to women prior to the elective cesarean section and our results indicated that it did not have significant effect on the metabolic profile of the women. Moreover, we consider peripartum depression a multifactorial disease^58^ suggesting that not all biochemical changes would be expected to be manifested in the blood plasma metabolic profile assessed by GC–MS metabolomics.

Despite these limitations, this study has provided valuable new insights about this pathophysiological condition, indicating season as a moderating factor, and pinpointed possible improvements in the case assessment process that are valuable for the study of this disease. The results obtained can thus form the basis for the design of future studies with larger cohorts.

## Supplementary information

Supplementary Information File

Normalized data set

## Data Availability

The datasets generated and/or analyzed during the current study are available from the corresponding author on reasonable request.
