# Supplementary material for: Blood plasma metabolic profiling of pregnant women with antenatal depressive symptoms
Source: Transl Psychiatry. 2019 Aug 23;9:204. doi: 10.1038/s41398-019-0546-y (PMC6707960; doi:10.1038/s41398-019-0546-y)
Supplement: Supplementary file 1 — Supplementary Information File [file 41398_2019_546_MOESM1_ESM.pdf]

SUPPLEMENTARY INFORMATION

Table of contents

Supplementary Table 1: List of all 50 samples, including seasonal belonging, EPDS score, time point for EPDS assessment, and age of participant. 2

Supplementary Table 2: The normalized metabolomic dataset of the study (provided as a separate file in Excel format).

Supplementary Table 3: The PC1, PC2 and PC3 loadings for each metabolite in the analyzed profiles as estimated from PCA. The difference in the abundance of the metabolites corresponding to large (in absolute values) loadings contributes more to the difference in the profiles over the corresponding principal component (PC). 4

Supplementary Figure 1: The PCA graph of the 47 samples indicating samples 25 and 33 as significantly different from the rest. 5

Supplementary Figure 2: The PCA graph with (A) and without (B) the names of the 45 metabolic profiles, color-coded as in Figure 1. Summer controls, summer cases, winter controls and winter cases are presented in yellow, red, light blue and dark blue, respectively.. 6

Supplementary Table 1: List of all 50 samples, including seasonal belonging, EPDS score, time point for EPDS assessment, and age of participant.

Sample number	Partus month and year	Season	EPDS score	EPDS time-point	Age at partus
Included samples					
1	Feb 2010	W	2	GW 32	37
2	Apr 2010	S	16	GW 32	35
3	May 2010	S	8	GW 32	35
4	Feb 2011	W	6	GW 32	33
5	Apr 2011	S	15	GW 32	36
6	Mar 2011	S	8	GW 32	34
7	Jun 2011	S	4	GW 32	30
8	Oct 2011	W	16	GW 32	30
9	Oct 2011	W	6	GW 32	29
10	Dec 2011	W	8	GW 32	35
11	Jan 2012	W	3	GW 32	33
12	Jan 2012	W	17	GW 32	33
13	Jan 2012	W	4	GW 32	35
14	Mar 2012	S	5	GW 32	34
15	May 2012	S	7	GW 32	37
16	Jul 2012	S	17	GW 32	34
17	Sep 2012	S	5	GW 32	32
18	Nov 2012	W	15	GW 32	37
19	Nov 2012	W	7	GW 32	31
20	Feb 2013	W	14	GW 32	28
21	Feb 2013	W	16	ECS	31
22	Mar 2013	W	5	ECS	33
23	Apr 2013	S	13	ECS	28
26	Aug 2013	S	1	GW 32	36
27	Nov 2013	W	2	GW 32	30
28	Nov 2013	W	8	GW 32	35
29	Dec 2013	W	3	GW 32	33
30	Feb 2014	W	12	ECS	34
31	Jan 2014	W	3	GW 32	35
32	Feb 2014	W	4	GW 32	35
34	Jun 2014	S	15	ECS	28
36	Mar 2014	W	1	ECS	33
37	Aug 2014	S	8	ECS	30
38	Mar 2014	S	12	ECS	34
39	Oct 2014	W	2	GW 32	36
40	Oct 2014	W	1	ECS	34
41	Oct 2014	W	14	GW 32	30
42	Nov 2014	W	3	ECS	35
43	Dec 2014	W	1	GW 32	31
44	Jan 2015	W	2	ECS	35

45	Mar 2015	W	2	ECS	37
46	Apr 2015	S	15	ECS	29
47	Dec 2014	W	21	ECS	27
48	May 2015	S	5	ECS	30
50	Sep 2015	S	7	GW 32	34
Excluded samples					
24	May 2013	S	1	GW 32	31
25	May 2013	S	17	GW 32	34
33	Feb 2014	W	15	GW 32	39
35	Mar 2014	W	4	ECS	34
49	Jul 2015	S	15	GW 32	34

S: summer; W: winter; EPDS: Edinburgh postnatal depression scale; GW: gestational week; ESC: Elective cesarean section

Supplementary Table 3: The PC1, PC2 and PC3 loadings for each metabolite in the analyzed profiles as estimated from PCA. The difference in the abundance of the metabolites corresponding to large (in absolute values) loadings contributes more to the difference in the profiles over the corresponding principal component (PC).

	**PC1**	**PC2**	**PC3**
lactate	1.01314	0.85054	0.77597
alanine 2TMS	-1.2962	0.85067	2.05116
Un_0012 (U_009)	0.13898	1.23444	0.29661
pyruvate	-0.4396	-1.22404	1.56073
2-hydroxybutanoic acid	0.60676	1.31676	0.23977
ethanolamine 3TMS	-1.17873	-0.56271	0.57709
glycerol	0.00621	1.48091	-0.38845
leucine 2TMS	-0.80938	-0.17512	2.61601
isoLeucine 2TMS	-0.76872	-0.12386	2.59537
serine 2TMS	-1.68557	0.18084	-0.63382
phosphate	-1.08823	-0.09056	-0.66973
glycerate	0.99615	-0.95983	-0.44855
urea	-0.53976	-0.49443	-1.06944
erythritol (putative)	0.18666	-0.44895	-1.12175
methyl benzoate	-0.62427	1.58088	-0.29754
Un_0089 (P1933,a_33)	1.22561	-0.28874	1.01047
Un_0245 (erythronate putative)	-0.23647	1.85981	-0.83029
aminomalonic acid	0.81823	0.36574	0.59066
threonate	-0.56111	0.63157	-0.80088
Un_0063 (A_068,u_032,a_39,x_2)	-0.52294	0.85911	0.26673
phenylalanine 2TMS	-1.72615	-0.3325	-0.70662
Un_0246 (RT:24.4,204,sugar pyranose)	1.79961	0.37254	-0.26793
arginine/ornithine 4TMS	-1.88119	-0.44913	0.7797
sorbitol	-1.0365	-0.56726	-1.22982
glutamine 3TMS	-1.63343	1.29013	-0.05402
gluconate	-0.02133	1.70172	-1.47411
myo-inositol	-0.67042	-0.60643	-1.32124
Un_0181 (P2922)	1.27085	-0.78347	0.12428
Un_0244 (RT:29.5,218)	-1.78509	0.36327	-0.3987
octadecanoic acid	-0.36203	1.88966	0.08485
linoleic acid	-0.56248	1.92498	-0.1088
cholesterol	-0.20482	0.48948	0.06553
glucose_total	1.58465	-0.01094	-0.04985
glutamate_effective	-0.85898	-1.09177	-0.67759
lysine_effective	-1.83733	0.8387	0.19768
threonine_effective	0.01926	-1.46205	-0.64631
valine_effective	-0.69944	-1.61704	-0.70917

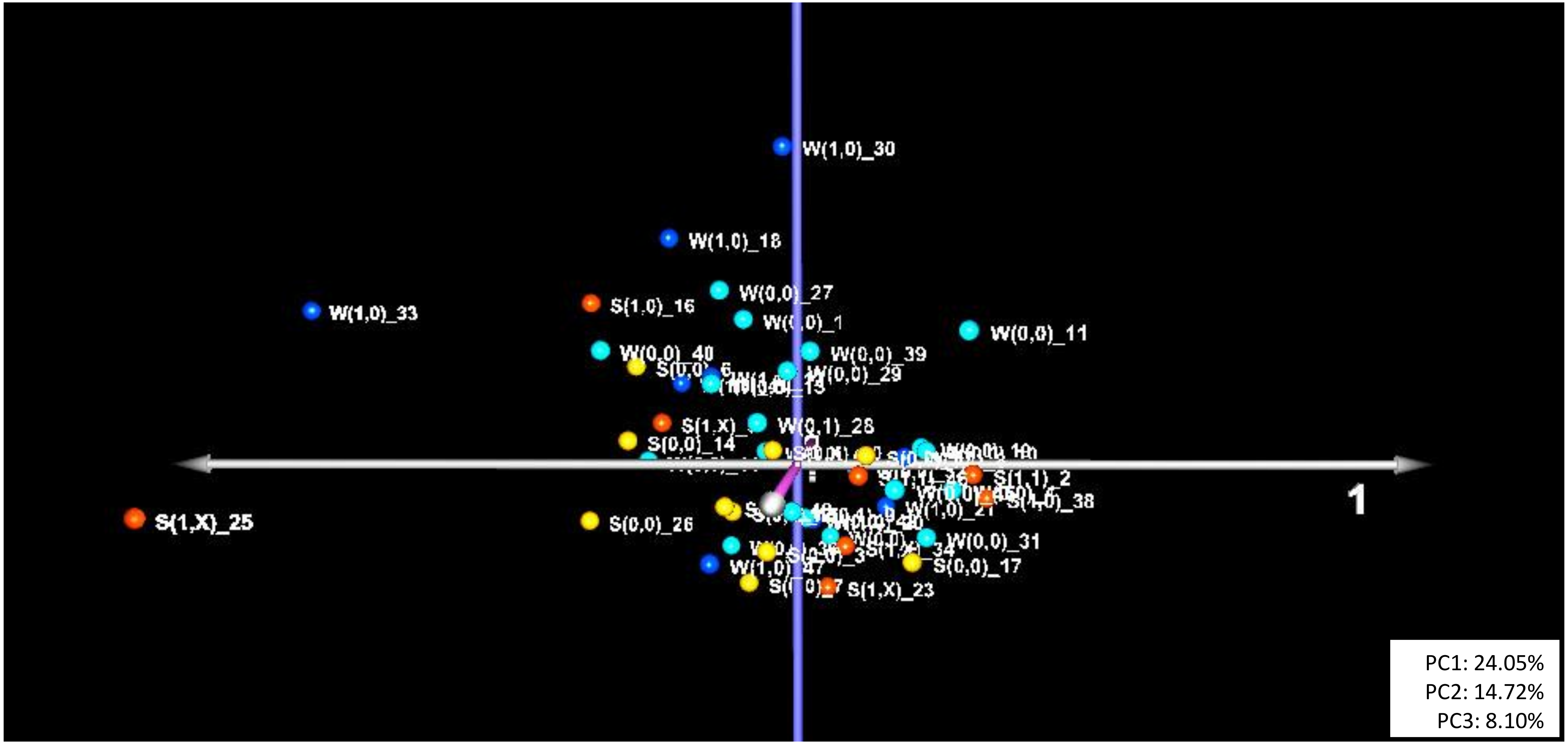

Supplementary Figure 1: The PCA graph of the 47 samples indicating samples 25 and 33 (left side of the graph) as significantly different from the rest.

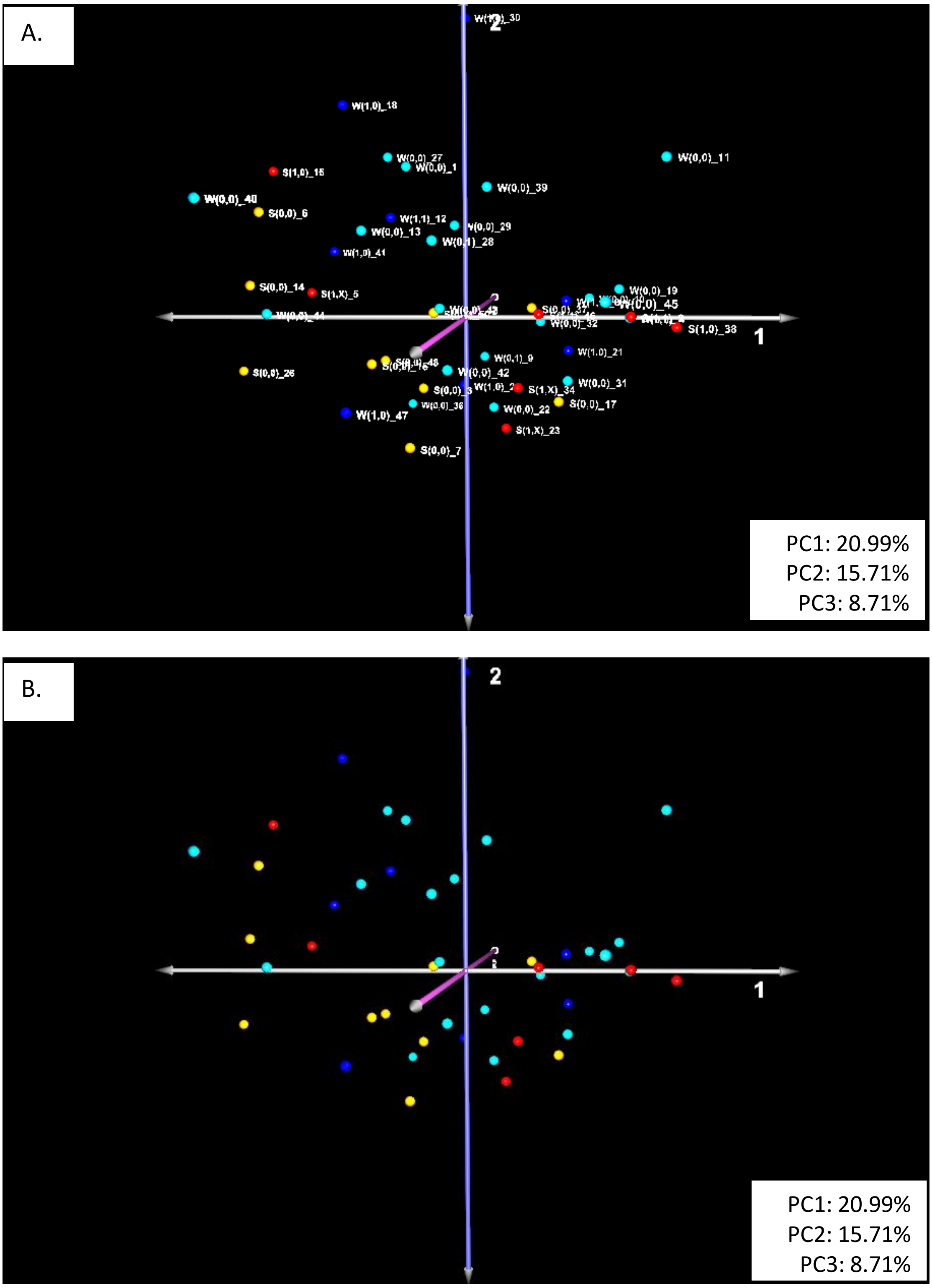

Supplementary Figure 2: The PCA graph with (A) and without (B) the names of the 45 metabolic profiles, color-coded as in Figure 1. Summer controls, summer cases, winter controls and winter cases are presented in yellow, red, light blue and dark blue, respectively.
