# Supplementary material for: Blood plasma metabolic profiling of pregnant women with antenatal depressive symptoms
Source: Transl Psychiatry. 2019 Aug 23;9:204. doi: 10.1038/s41398-019-0546-y (PMC6707960; doi:10.1038/s41398-019-0546-y)
Supplement: Supplementary file 2 — Normalized data set [file 41398_2019_546_MOESM2_ESM.pdf]

Peak Name
lactate 2TMS
alanine 2TMS
Un_0012 (U_009; alpha-hydroxyisobutyrate putative)
pyruvate 2TMS
2-hydroxybutanoic acid 2TMS
ethanolamine 3TMS
glycerol 3TMS
leucine 2TMS
isoLeucine 2TMS
serine 2TMS
phosphate 4TMS
glycerate
urea 2TMS
erythritol 4TMS (putative)
3-methyl benzoate 1TMS
Un_0089 (P1933, a_33)
Un_0245 (erythronate putative)
aminomalonic acid 3TMS
threonate 3TMS
Un_0063 (A_068, u_032, a_39, x_2)
phenylalanine 2TMS
paracetamol 2TMS
Un_0246 (RT:24.4, 204, sugar pyranose)
arginine/ornithine 4TMS
sorbitol 6TMS
glutamine 3TMS
gluconate 6TMS
myo-Inositol 6TMS
Un_0181 (P2922)
Un_0244 (RT:29.5, 218)
octadecanoic acid 1TMS
linoleic acid 1TMS
cholesterol 1TMS
glucose_total
glutamate_effective
lysine_effective
threonine_effective
valine_effective

Note: For the unknown metabolites, the number in the ME
***Chemical Category** refers to the categorization with respe

Metabolite Name	RT	Quan Ion	Chem. Cat.*	min
lactate	7.04	117	1	18.33
alanine 2TMS	7.86	116	3	0.45
Un_0012 (U_009; alpha-hydroxyisobutyrate putative)	8.94	204	1	0.81
pyruvate	9.43	174	1	0.29
2-hydroxybutanoic acid	10.03	191+117	1	0.78
ethanolamine 3TMS	11.23	174	3	0.14
glycerol	12.03	205	1	1.32
leucine 2TMS	12.65	158	3	0.12
isoLeucine 2TMS	13.3	158	3	0.06
serine 2TMS	14.16	116	3	0.32
phosphate	14.55	387	1	1.26
glycerate	14.85	292	1	0.17
urea	14.93	189	1	65.92
erythritol (putative)	17.64	217	1	0.33
3-methyl benzoate	18.4	193	1	0.36
Un_0089 (P1933, a_33)	19.21	232	1	0.64
Un_0245 (erythronate putative)	19.6	292	1	0.35
aminomalonic acid	19.61	218	1	0.10
threonate	20.29	292	1	0.36
Un_0063 (A_068, u_032, a_39, x_2)	20.92	263	1	0.54
phenylalanine 2TMS	23.65	218	3	1.30
paracetamol	23.95	206	1	0.00
Un_0246 (RT:24.4, 204, sugar pyranose)	24.37	204	1	0.13
arginine/ornithine 4TMS	25.03	142	3	0.33
sorbitol	25.91	319	1	3.56
glutamine 3TMS	26.91	156	3	0.91
gluconate	27.86	333	1	0.29
myo-inositol	28.95	305	1	3.16
Un_0181 (P2922)	29.05	361	1	1.19
Un_0244 (RT:29.5, 218)	29.5	218	1	0.77
octadecanoic acid	34.13	117	1	6.52
linoleic acid	34.42	337	1	0.77
cholesterol	48.49	368	1	10.46
glucose_total			2	318.51
glutamate_effective			3	9.31
lysine_effective			3	0.97
threonine_effective			3	2.48
valine_effective			3	1.94

SBL database is provided and in parenthesis previously assigned by our group annotations for the same peak
ct to the number and type of derivatives for a metabolite as defined in Kanani and Klapa, 2007.

max	mean	median	W(0,0)_1	S(1,1)_2	S(0,0)_3	W(0,0)_4	S(1,X)_5	S(0,0)_6
268.81	55.34	46.68	48.60	42.94	67.09	67.24	31.76	39.70
14.39	3.83	3.23	5.27	1.12	2.54	0.52	4.50	6.00
4.38	1.85	1.60	1.36	1.60	1.50	1.08	2.39	1.70
3.86	1.66	1.48	0.60	1.10	2.27	0.33	1.12	1.82
24.43	4.09	2.75	1.81	4.60	2.98	2.60	6.07	2.94
1.37	0.46	0.40	0.46	0.31	0.26	0.27	0.65	0.64
6.89	3.51	3.41	4.38	3.58	1.72	3.55	2.67	6.44
3.67	1.27	1.10	0.94	0.33	1.07	0.21	1.14	1.70
2.56	0.74	0.61	0.46	0.32	0.26	0.15	0.61	0.96
3.35	1.43	1.32	0.81	1.04	1.75	0.80	2.08	1.58
21.12	7.06	6.78	7.32	1.53	9.28	1.95	7.70	8.84
1.51	0.75	0.73	0.44	0.40	0.45	0.51	0.48	0.48
268.49	154.24	148.93	140.56	156.10	128.93	102.04	199.88	148.93
2.14	0.59	0.54	0.57	0.54	0.39	0.62	0.52	0.55
0.92	0.62	0.61	0.78	0.57	0.50	0.42	0.71	0.68
6.92	2.71	2.43	1.74	3.47	6.92	3.09	2.11	2.71
4.99	1.18	0.77	1.98	0.69	0.56	0.70	1.44	0.60
2.18	0.95	0.91	0.77	0.98	0.49	1.61	0.95	1.36
1.97	1.14	1.14	1.32	0.60	1.83	0.36	0.96	1.63
2.18	1.19	1.12	1.61	0.80	0.77	0.78	1.59	1.36
5.40	2.96	2.80	3.11	1.76	2.81	2.55	4.03	3.53
3.94	0.18	0.00	0.00	3.94	0.00	0.00	0.00	0.00
7.78	2.46	2.15	2.18	4.34	1.69	3.44	2.98	2.04
7.12	1.11	0.86	0.82	0.53	1.54	Missing	1.27	1.55
12.07	8.17	7.99	6.21	3.56	11.54	4.52	9.79	7.99
31.99	6.14	4.24	7.57	2.37	3.03	3.61	9.66	14.92
1.64	0.62	0.53	0.93	0.29	0.42	0.50	0.69	0.44
11.31	5.73	5.38	5.61	3.16	4.28	4.02	6.19	6.27
5.25	3.18	3.23	1.48	4.74	2.39	3.06	3.43	3.14
10.75	3.03	2.84	3.39	1.19	2.78	1.18	4.46	4.33
29.39	14.88	14.82	16.73	14.11	10.35	11.29	16.13	17.53
3.61	1.85	1.67	2.12	1.48	1.31	1.03	2.05	2.42
107.56	52.35	49.76	38.38	10.46	46.73	45.43	47.25	43.50
1211.52	738.13	733.18	715.91	765.67	630.08	608.25	768.74	526.11
50.46	18.86	17.51	13.07	14.60	29.27	10.68	24.25	16.12
11.36	4.66	3.50	5.69	2.17	3.50	1.26	7.36	11.36
10.61	5.97	5.58	3.01	4.41	5.71	4.96	6.28	5.41
10.44	5.74	5.63	3.15	3.63	6.26	4.26	9.91	4.70

that have been included in published reports and publications.

S(0,0)_7	W(1,1)_8	W(0,1)_9	W(0,0)_10	W(0,0)_11	W(1,1)_12	W(0,0)_13	S(0,0)_14
55.61	45.01	36.39	47.10	86.77	67.81	38.41	34.72
4.92	1.31	1.21	0.82	1.63	4.52	8.40	7.58
1.55	1.15	2.54	1.56	3.98	2.57	0.94	0.93
2.28	0.93	1.38	0.29	1.27	1.34	1.55	1.99
3.11	1.71	10.05	3.12	24.43	6.65	0.78	2.38
0.85	0.28	0.45	0.16	0.26	0.32	0.71	0.51
2.52	4.63	4.39	2.44	2.34	4.62	4.04	4.32
2.55	0.86	0.40	0.15	0.52	0.81	1.82	1.72
1.45	0.50	Missing	0.16	0.35	0.43	1.12	0.89
0.92	0.90	1.26	1.27	0.71	1.54	1.25	1.68
8.22	3.88	13.98	3.32	2.59	11.06	6.78	13.07
0.50	0.85	1.03	0.86	0.72	0.76	0.36	0.38
190.46	104.18	151.82	104.47	145.07	162.57	83.84	136.20
0.67	0.54	2.14	0.53	0.50	0.67	0.58	0.64
0.53	0.59	0.49	0.44	0.68	0.67	0.67	0.64
2.09	1.63	5.49	2.23	2.68	1.77	3.33	0.95
0.57	0.73	0.71	0.66	0.86	1.07	1.93	0.62
0.37	1.35	0.95	0.81	0.59	0.66	1.28	0.48
0.42	0.90	1.40	0.72	0.82	1.37	1.33	1.17
0.68	0.79	1.04	0.75	0.92	1.38	1.08	1.01
3.25	2.13	3.80	2.21	1.33	3.51	2.60	3.37
0.00	0.00	0.00	2.08	0.00	0.00	0.00	0.00
1.88	4.11	3.80	6.07	5.22	2.41	2.93	0.80
0.99	0.53	0.64	0.49	0.33	1.09	0.86	1.90
9.13	7.15	7.80	12.07	6.36	6.79	8.96	7.76
1.72	4.24	4.16	4.50	2.85	7.50	9.05	10.48
0.43	0.46	0.45	1.33	0.53	0.65	0.94	0.55
6.46	5.36	6.36	4.84	4.94	5.99	6.89	5.38
4.36	3.99	4.51	3.59	3.18	1.68	2.17	2.36
2.24	1.77	3.57	2.43	1.74	3.21	2.90	4.54
8.15	13.28	15.17	11.71	22.50	17.16	15.82	15.68
1.10	1.51	1.61	1.34	2.33	2.27	1.80	1.73
44.88	39.56	49.76	52.71	58.29	55.13	51.79	39.20
679.93	646.65	873.64	1019.25	1211.52	824.44	630.03	607.35
21.09	12.49	26.06	16.92	9.31	15.32	19.10	15.58
3.25	2.58	4.37	2.34	3.31	7.34	5.18	8.44
7.51	3.97	5.89	4.77	4.19	5.61	2.93	5.00
6.51	4.06	7.99	4.83	4.08	6.47	4.24	6.45

S(0,0)_15	S(1,0)_16	S(0,0)_17	W(1,0)_18	W(0,0)_19	W(1,0)_20	W(1,0)_21	W(0,0)_22
43.27	69.45	28.95	45.85	77.43	29.99	63.13	29.10
3.91	6.00	1.10	4.20	2.40	0.98	0.45	1.34
1.61	4.38	0.81	1.90	1.23	1.97	1.10	1.45
3.07	1.83	2.53	0.29	1.42	3.86	0.64	3.35
1.83	7.51	1.65	3.33	2.31	2.64	3.09	0.93
0.55	0.60	0.24	0.33	0.39	Missing	Missing	0.29
2.47	6.89	1.93	3.24	6.31	2.10	2.88	2.99
1.74	1.55	0.61	1.23	1.04	0.27	0.28	0.54
0.91	0.95	0.33	0.65	0.44	0.12	0.06	0.30
1.32	1.66	0.99	2.11	0.32	1.33	0.92	0.77
7.80	9.85	8.80	4.71	1.94	6.03	2.93	4.50
0.49	1.10	0.55	0.17	1.19	0.73	1.50	0.56
169.96	268.49	81.98	141.43	145.19	138.67	156.26	259.66
0.51	0.64	0.38	0.46	0.69	0.43	0.72	0.57
0.56	0.74	0.44	0.80	0.82	0.62	0.60	0.56
1.72	3.02	3.82	0.76	2.43	2.44	2.33	2.77
0.50	1.40	0.55	4.99	1.14	0.50	1.35	0.50
0.62	1.03	1.10	0.84	1.21	0.65	0.59	0.91
0.91	1.14	0.71	1.97	1.68	1.55	1.00	0.89
0.98	1.63	0.79	1.44	1.12	1.32	1.03	1.35
3.04	4.49	2.34	2.97	2.58	2.80	2.55	2.67
0.00	1.93	0.00	0.00	0.00	0.00	0.00	0.00
1.97	2.29	3.04	0.43	7.78	1.43	3.42	1.14
1.44	1.03	0.81	0.95	0.36	1.02	Missing	0.70
7.98	10.62	5.18	7.72	8.97	10.49	7.62	9.78
5.64	12.13	2.06	8.78	2.34	1.32	3.34	1.06
0.54	0.77	0.46	1.16	0.61	0.39	0.72	0.39
5.93	8.62	5.04	4.78	5.67	5.96	6.80	7.07
3.51	2.46	3.40	2.94	4.56	4.52	4.38	5.25
2.93	4.86	2.17	3.50	2.96	1.64	2.61	1.69
13.10	29.39	6.52	19.30	17.07	11.56	9.19	12.81
1.46	3.17	0.77	2.66	1.19	1.66	1.50	1.55
38.57	71.86	35.33	107.56	46.30	66.38	58.16	66.75
604.05	733.92	867.79	494.22	1111.96	765.78	939.80	591.76
16.68	22.82	15.58	17.51	18.72	21.19	17.91	15.34
5.86	10.35	2.56	5.08	0.97	3.76	2.46	3.32
5.81	8.79	3.32	4.85	9.88	5.20	7.60	10.18
5.63	7.16	5.09	3.58	4.22	6.40	5.96	5.06

S(1,X)_23	S(1,X)_25	S(0,0)_26	W(0,0)_27	W(0,1)_28	W(0,0)_29	W(1,0)_30	W(0,0)_31
57.42	268.81	46.68	43.56	86.08	47.60	57.24	18.33
0.60	13.98	5.04	3.50	7.61	0.85	5.08	0.83
1.61	1.50	1.72	2.65	3.18	2.49	2.11	1.08
1.83	2.17	2.81	0.33	1.29	1.11	0.38	1.77
1.36	2.80	1.77	9.66	5.41	10.25	7.42	2.16
0.49	0.55	0.55	0.35	0.44	0.32	0.31	0.14
2.52	2.64	2.45	5.55	3.92	3.39	5.51	1.62
0.46	3.31	1.89	1.26	1.69	0.54	0.45	0.32
0.47	2.56	1.04	0.79	0.89	0.29	0.22	0.19
0.79	3.35	2.07	2.20	1.34	1.95	0.99	0.82
2.04	21.12	8.24	7.82	8.84	4.82	10.84	4.92
0.74	0.79	1.05	1.07	0.57	0.81	0.22	0.64
221.63	165.62	186.89	130.37	108.64	161.65	159.73	65.92
0.67	1.05	0.76	0.65	0.63	0.38	0.55	0.33
0.39	0.71	0.64	0.72	0.65	0.66	0.89	0.36
2.60	1.08	1.37	1.68	3.06	3.05	3.75	1.52
0.77	1.16	0.91	2.75	0.97	0.42	4.03	0.35
0.42	0.86	1.00	0.70	0.53	1.47	1.12	0.49
1.01	1.97	1.61	1.63	0.74	1.46	1.44	1.06
0.54	1.55	0.98	1.38	1.02	1.03	1.47	0.63
3.08	5.09	4.07	2.65	3.24	2.53	2.57	1.76
0.00	0.00	0.00	0.00	0.00	0.00	0.36	0.00
2.19	0.43	0.88	1.85	2.08	2.15	4.33	1.34
0.60	7.12	2.33	0.90	0.72	0.76	0.43	0.48
7.61	5.08	9.40	10.23	5.23	6.00	8.33	7.12
3.43	20.72	10.55	9.15	4.68	5.42	8.09	0.91
0.56	0.81	0.57	0.90	0.53	0.55	1.64	0.38
5.15	8.86	9.52	4.90	4.37	6.15	5.32	4.61
2.34	1.19	2.04	2.97	2.28	1.60	3.23	3.35
2.14	6.10	4.35	2.81	3.66	2.42	3.04	1.31
7.83	17.56	12.51	21.07	14.58	18.88	25.21	7.30
0.88	1.92	1.67	2.73	1.73	3.15	3.61	0.95
36.28	73.33	46.12	59.98	36.41	45.85	54.69	36.56
618.56	318.51	673.43	714.46	786.97	527.43	818.57	579.62
20.29	50.46	20.68	22.09	14.13	17.50	14.93	11.59
2.67	10.27	8.29	5.98	4.40	4.11	5.63	2.25
10.61	9.11	8.50	4.55	4.11	3.58	2.54	2.48
6.97	10.44	8.98	5.03	6.24	6.28	1.94	3.40

W(0,0)_32	W(1,0)_33	S(1,X)_34	W(0,0)_36	S(0,0)_37	S(1,0)_38	W(0,0)_39	W(0,0)_40
57.11	54.39	39.08	40.52	39.49	63.05	76.91	42.10
1.52	14.39	2.69	0.65	0.59	1.26	5.11	6.85
1.16	2.14	0.93	1.27	1.51	1.86	4.18	2.14
1.17	1.46	1.48	1.10	2.29	1.25	2.85	2.43
2.75	4.03	1.95	2.10	2.40	2.66	7.50	6.30
0.30	1.37	0.26	0.91	0.21	Missing	0.24	0.69
3.77	3.80	3.10	2.14	4.85	4.00	3.97	4.62
0.95	2.05	1.82	0.38	0.12	1.71	0.50	3.67
0.42	1.09	1.06	0.21	Missing	Missing	0.54	2.01
1.55	3.13	1.05	2.18	0.88	Missing	1.06	2.43
4.84	14.69	9.39	11.55	11.75	1.26	7.49	5.27
0.85	0.42	1.51	1.32	1.01	1.16	0.46	0.61
194.66	171.24	177.45	207.64	135.62	147.42	140.29	155.63
0.59	0.77	0.59	0.53	0.48	0.41	0.46	0.33
0.62	0.85	0.50	0.51	0.55	Missing	0.56	0.82
3.14	1.23	4.42	1.27	5.17	5.12	2.51	2.27
0.86	1.07	1.51	1.41	0.75	1.16	4.07	0.57
1.88	0.98	1.39	0.35	1.24	1.61	0.85	1.24
0.58	1.18	1.23	1.05	0.99	0.79	0.94	0.74
1.42	2.18	1.46	1.15	1.61	0.93	1.16	1.88
3.04	5.40	2.66	3.14	2.56	1.30	2.09	3.52
0.00	0.00	0.00	0.00	0.00	0.00	0.00	0.00
4.45	0.60	3.66	2.27	2.19	3.65	0.87	1.05
0.39	2.93	0.69	0.68	0.64	Missing	0.40	1.62
7.50	10.39	7.05	10.69	7.21	5.34	8.70	8.20
3.18	31.99	2.72	3.85	1.68	Missing	4.24	11.77
0.64	1.02	0.45	0.77	0.40	0.89	0.67	0.43
6.65	11.31	5.62	6.51	4.85	5.38	3.73	4.00
4.93	2.68	4.07	1.76	3.49	3.57	2.47	3.36
2.60	10.75	3.01	2.90	1.86	0.77	2.07	4.91
12.75	19.14	9.01	8.35	15.17	12.59	14.82	27.01
2.26	2.42	1.36	1.32	2.54	0.91	1.94	3.26
69.58	66.95	53.13	37.20	69.63	35.68	38.45	67.46
951.93	474.02	888.71	833.56	756.70	969.74	614.28	750.02
17.55	24.27	19.84	22.76	16.43	12.07	16.84	21.14
3.41	10.97	3.23	3.45	3.24	1.36	3.28	7.57
7.52	8.47	8.33	10.05	5.23	7.13	3.97	6.35
7.02	4.41	6.22	9.13	5.81	5.07	3.77	6.07

W(1,0)_41	W(0,0)_42	W(0,0)_43	W(0,0)_44	W(0,0)_45	S(1,1)_46	W(1,0)_47	S(0,0)_48
43.00	52.63	63.54	39.76	79.22	66.69	42.36	42.10
2.28	5.78	3.23	6.31	6.74	2.10	5.99	3.35
1.58	1.52	2.58	1.47	2.49	2.14	1.26	1.32
0.82	2.43	0.95	1.76	1.78	2.73	3.21	0.97
1.69	1.56	1.02	1.15	7.42	2.58	2.65	3.30
0.41	0.72	0.69	0.40	0.35	Missing	0.53	0.39
3.93	2.39	3.68	2.39	3.27	3.41	1.32	2.50
0.70	1.51	2.29	2.75	3.48	Missing	2.40	1.39
0.34	0.88	1.00	1.38	2.13	Missing	1.43	0.85
2.14	1.59	1.09	1.48	0.62	1.53	1.31	1.73
6.21	3.85	1.27	5.36	4.34	6.78	6.63	9.60
0.38	0.75	0.87	0.78	1.41	1.25	0.46	0.71
217.73	163.96	238.15	183.55	112.59	114.64	120.05	108.38
0.52	0.55	0.49	0.49	0.48	0.51	0.38	0.53
0.77	0.61	0.92	0.70	0.53	0.54	0.40	0.51
0.64	4.55	2.22	1.48	6.52	3.73	1.73	1.55
2.19	0.60	1.83	0.92	0.77	0.72	0.49	0.47
0.57	2.03	2.18	0.68	1.27	1.39	0.10	0.16
1.06	0.85	1.46	1.18	1.38	1.46	0.80	1.14
0.92	1.05	1.45	1.32	1.45	0.95	0.98	1.14
4.33	3.11	3.37	4.01	1.93	2.06	2.77	2.69
0.00	0.00	0.00	0.00	0.00	0.00	0.00	0.00
0.49	1.95	2.06	1.14	5.19	3.31	0.13	0.23
1.07	1.09	0.86	1.96	Missing	0.46	1.09	0.91
10.28	9.21	7.60	10.94	5.25	8.74	8.67	11.16
8.11	4.50	4.31	8.79	2.20	1.72	1.87	3.57
0.76	0.42	0.47	0.52	0.45	0.51	0.38	0.41
6.18	4.56	5.98	5.34	5.30	4.51	4.20	5.59
2.03	4.48	4.17	2.93	4.18	3.53	1.26	2.54
4.45	2.84	2.86	4.53	1.36	1.56	2.73	2.87
14.84	11.44	12.34	15.29	15.01	20.38	9.49	14.00
2.07	1.41	1.61	1.67	2.12	2.15	1.26	1.52
27.02	39.45	42.28	64.84	69.93	51.84	58.46	67.81
650.35	733.18	715.76	599.74	1112.72	797.41	610.76	753.78
16.91	17.13	17.44	17.76	16.81	21.13	20.90	24.84
5.61	3.10	3.06	6.66	2.34	3.20	4.83	4.21
5.58	5.45	8.48	6.99	6.13	4.64	5.28	4.41
5.15	4.87	7.92	4.92	5.40	6.30	5.70	7.77

S(0,X)_50
36.79
2.85
1.64
2.62
3.90
0.42
3.61
1.36
0.67
1.60
6.80
0.98
143.16
0.56
0.53
1.97
0.55
0.54
1.24
2.13
2.51
0.00
1.93
0.94
9.90
2.73
0.44
4.94
3.75
2.35
18.21
2.29
97.51
825.17
21.43
3.33
5.88
5.26
